# Supplementary material for: Soluble Heparin and Heparan Sulfate Glycosaminoglycans Interfere with Sonic Hedgehog Solubilization and Receptor Binding
Source: Molecules. 2019 Apr 23;24(8):1607. doi: 10.3390/molecules24081607 (PMC6526471; doi:10.3390/molecules24081607)
Supplement: Supplementary file 1 [file molecules-24-01607-s001.pdf]

Supplemental materials for

Soluble Heparin and Heparan Sulfate Glycosaminoglycans Interfere with Sonic Hedgehog Solubilization and Receptor Binding

Dominique Manikowski, Petra Jakobs, Hamodah Jboor & Kay Grobe

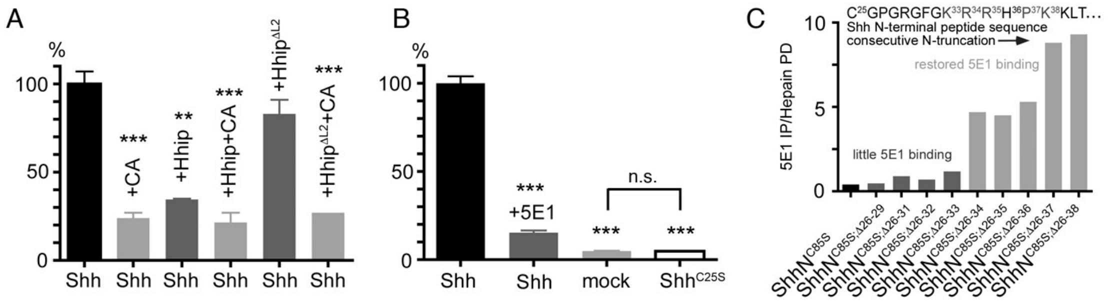

Supplementary Figure S1. Modulated Ptc-receptor binding of Shh. (**A**) Shh–Ptc binding is physiologically modulated by competing Shh–Hhip interactions. Deletion of a Hhip peptide loop known to interact with the Shh zinc coordination site that also serves as the Ptc interaction site ($Hhip^{\Delta L2}$) impairs Shh binding and restores most signaling activity. Cyclopamine (CA)—an antagonist acting downstream of Ptc—serves as a control for Shh-specific induction of C3H10T1/2 differentiation. (**B**) Shh–Ptc interactions are also blocked by competing unprocessed N-terminal Shh^{C25S} peptides. The monoclonal antibody 5E1 blocks the same site. (**C**) Unprocessed N-terminal peptides block Shh–Ptc interactions. Unlike dual-lipidated Shh, $ShhN^{C25S}$ is an artificial, non-lipidated Shh variant that undergoes simple secretion without terminal processing. Proteins in $ShhN^{C25S}$ conditioned media and media containing gradually N-terminally truncated $ShhN^{C25S}$ variants were pulled down with heparin agarose (which binds all forms) or immunoprecipitated with 5E1-coupled ProteinA-agarose (which binds the exposed accessible Ptc interaction site) and analyzed by immunoblotting. Ratios between the 5E1-immunoprecipitated material and the proteins pulled down by heparin (from the same supernatants) are shown. Note that artificial N-terminal truncations restore 5E1 MoAb binding to the Ptc binding site.

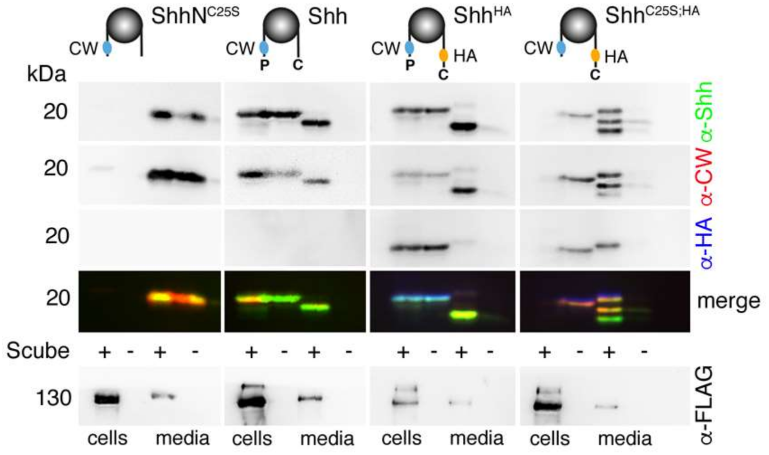

Supplementary Figure S2. Proteolytic processing of N-terminal peptides during Shh release. In contrast to dual-lipidated membrane-tethered Shh, artificial ShhN^{C25S} is not lipidated and therefore undergoes direct secretion. C-terminally hemagglutinin (HA)-tagged Shh^{HA} and non-palmitoylated, but cholesteroylated Shh$^{C25A;HA}$ were also analyzed. The latter two proteins carry an extended C-terminal membrane anchor (N^{190}SVAAKSG-*YPYDVPDYA*-G^{198} (G^{198} represents the cholesterol-modified glycine; underlined italicized letters represent the tag) [12]). Proteins in the cellular (cells) and corresponding soluble fractions (media) were analyzed by immunoblotting. α-CW antibodies raised against the CW peptide K^{33}RRHPKK^{39} detected N-terminal processing of released proteins, α-HA antibodies detected the C-terminal tag, and polyclonal α-Shh antibodies detected full-length and truncated proteins on the same (stripped) blot. To better demonstrate Shh processing during release, we inverted and colored the gray scale blots (green: α-Shh signal, red: α-CW signal, blue: α-HA signal). Signals of increased electrophoretic mobility therefore denote C-processed/N-unprocessed soluble proteins, and most mobile green signals confirm the removal of N- and C-terminal peptides. Scube2 increased the release of all lipidated Shh forms (compare lipidated proteins + Scube released into the media with proteins expressed in the absence of Scube2) and converted cellular Shh and Shh^{HA} into truncated soluble morphogens. This is indicated by an electrophoretic size shift and lack of α-CW and all α-HA antibody reactivity. Cell-surface-associated Shh$^{C25A;HA}$ was completely C-terminally processed (yellow band, compare with Shh^{HA}) and also underwent partial N-terminal processing (green band) [20].

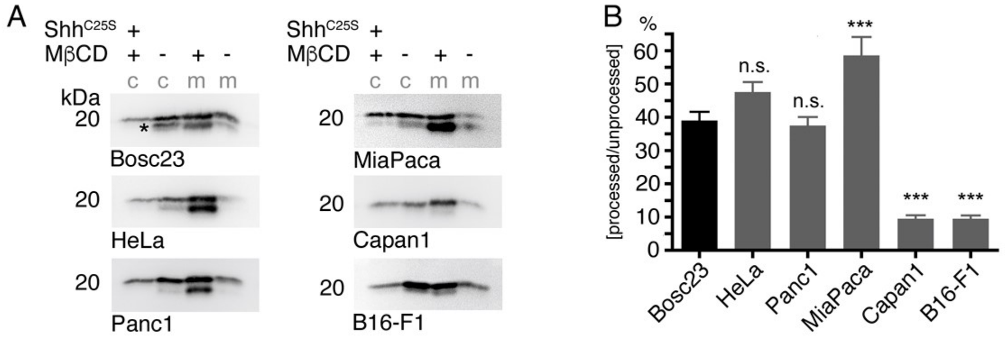

Supplementary Figure S3. Proteolytic Shh truncation at the surface of cancer cells. (**A**) In Bosc23 cells in the presence of the unspecific sheddase-activator methyl-β-cyclodextrin, a substantial fraction of soluble Shh^{C25S} is released in N-terminally unprocessed form. This is indicated by its unchanged electrophoretic mobility, but proteolytic CW processing truncates the remaining fraction and increases its electrophoretic mobility (bottom band, asterisk). Shh^{C25S} N-truncation was also observed in HeLa (adenocarcinoma) (n = 12 assays), Panc1 (pancreatic carcinoma) (n = 6), and MiaPaCa (pancreatic

carcinoma) cells (n = 2). By contrast, Capan1 (pancreatic carcinoma derived from metastatic site in the liver) (n = 3) and B16F6 (mouse melanoma) cells (n = 2) produced only unprocessed Hh^{C25S}. (**B**) Cellular capacities to N-process Hh^{C25S}. Bosc23: 39% ± 6% of soluble proteins were N-processed, n = 6; HeLa: 48% ± 7% of soluble proteins were N-processed, $p > 0.05$ compared with Bosc23, n = 5; Panc1: 38% ± 6% of soluble proteins were N-processed, $p > 0.05$, n = 6; MiaPaCa: 59% ± 12% of soluble proteins were N-processed, $p < 0.001$, n = 5; Capan1: 9% ± 2% of soluble proteins were N-processed, $p < 0.001$, n = 6; B16-F1: 9% ± 2% of soluble proteins were N-processed, $p < 0.001$, n = 6. N-terminal Shh processing and release may therefore contribute to paracrine Shh signaling in a variable, cancer cell-type specific manner [34, 35].

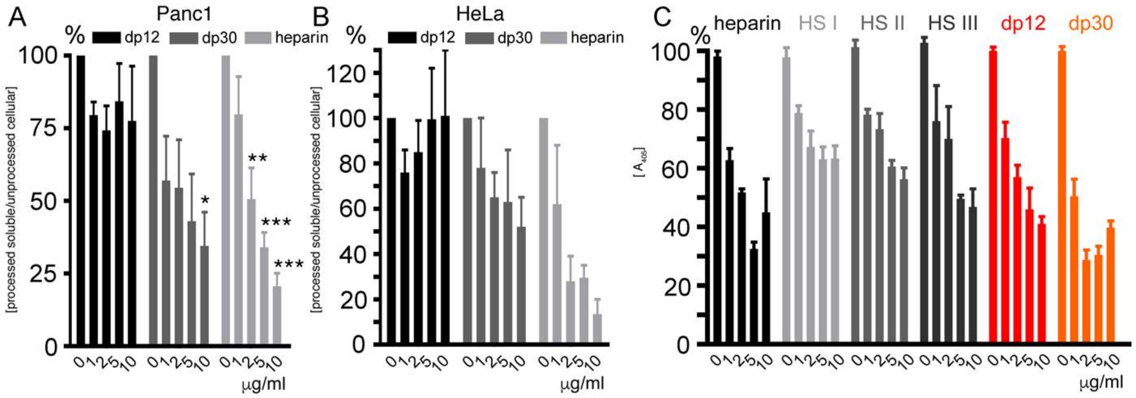

Supplementary Figure S4. (**A**) Dose-dependent inhibition of Shh solubilization from Panc1 and HeLa cells by heparin oligosaccharides of variable length. dp12: 12 sugar units, dp30: 30 sugar units. Shh released in the absence of glycosaminoglycans was always set to 100%. 1 µg/mL heparin: 80% ± 29%, 2 µg/mL heparin: 51% ± 24%, 5 µg/mL heparin: 34% ± 11%, 10 µg/mL heparin: 21 ± 10, n = 5 in all cases. 1 µg/mL dp30: 57% ± 31%, 2 µg/mL dp30: 54% ± 33%, 5 µg/mL dp30: 43% ± 32%, 10 µg/mL dp30: 34 ± 23, n = 4 in all cases. * $p < 0.05$, ** $p < 0.01$, *** $p < 0.001$, n = 4–5. dp12: all n.s. ($p > 0.05$). (**B**) Shh release from HeLa cells in the presence of increasing amounts of dp12, dp30, and heparin. $p > 0.05$ in all cases, n = 2. (**C**) Dose-dependent inhibition of Shh-induced C3H10T1/2 precursor cell differentiation by variably sulfated glycosaminoglycans (HSI-III and heparin) or by heparin oligosaccharides of variable length. * $p < 0.05$, ** $p < 0.01$, *** $p < 0.001$.

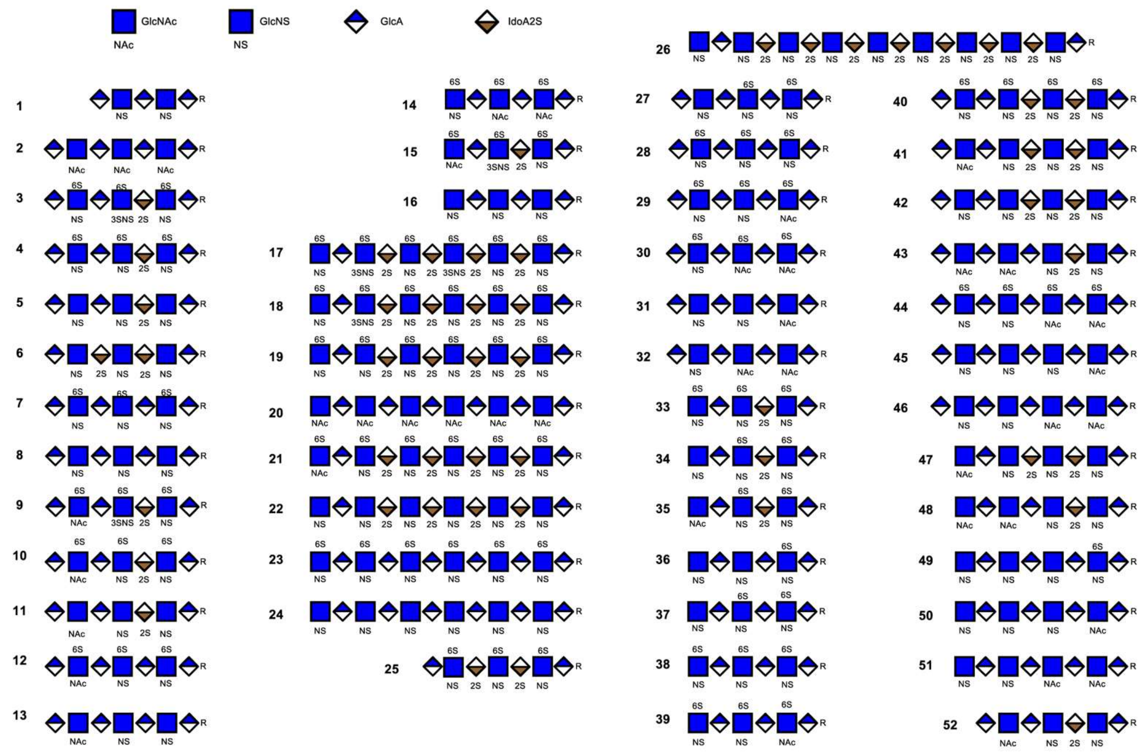

Supplementary Figure S5. Schematic of spotted glycan structures analyzed in this study. R denotes the reducing end of the oligosaccharide coupled to the chip surface.
